# The Role of the Western Diet on Atopic Dermatitis: Our Experience and Review of the Current Literature

**DOI:** 10.3390/nu15183896

**Published:** 2023-09-07

**Authors:** Proietti Ilaria, Tolino Ersilia, Bernardini Nicoletta, Trovato Federica, Vizzaccaro Andrea, Skroza Nevena, Potenza Concetta

**Affiliations:** Unit of Dermatology, Department of Medical-Surgical Science and Biotechnologies, Sapienza University of Rome, A. Fiorini Hospital, 04019 Terracina, Latina, Italy; ersiliatolino@gmail.com (T.E.); nicoletta.bernardini@uniroma1.it (B.N.); andrea.vizzaccaro@uniroma1.it (V.A.); nevena.skroza@uniroma1.it (S.N.); concetta.potenza@uniroma1.it (P.C.)

**Keywords:** atopic dermatitis, diet, Mediterranean diet, Western diet

## Abstract

The correlation between health and diet has always been a subject of interest in the field of dermatology and medicine in general. However, studies in the literature are still scarce, and need further investigation in the field of inflammatory skin diseases. In this paper, we report a case of a patient with atopic dermatitis whose complete recovery occurred only after combining dupilumab therapy with a Mediterranean diet regimen.

## 1. Introduction

Global dietary patterns have changed, affecting people’s health globally. These changes have both had a positive effect, introducing health-promoting eating habits, and negative effects, mainly in health in low- and middle-income countries. In 2013, the Global Panel on Agriculture and Food Systems for Nutrition (GloPan) convened in order to classify foods into two categories, “healthy foods” and “unhealthy foods”. “Healthy foods” were included in Panel A, and were defined as foods that should be the foundation of a balanced diet. “Unhealthy foods” were included in Panel B, and were defined as foods that should be consumed in moderation. As part of the meeting, an analysis was conducted on the consumption of different foods in different regions, which revealed higher fruit consumption in higher income regions, with lower vegetable consumption. Seafood consumption was found to be relatively low worldwide except in Southeast Asia; instead, dairy consumption was higher in North America and Europe. Regarding Panel B foods, red meat consumption was similar in East Asia, Latin America, North America, and Europe; Trans Fatty Acid (TFA) consumption was higher in South Asia; sugar-sweetened beverages (SSBs) were more popular in Latin and North America [1]. In recent decades, there has been a global increase in the prevalence of Western-style diets (WDs) in Europe. These diets include processed foods, “junk food”, convenience products, sugary drinks, and animal products which have reduced fiber, vitamin, and mineral content. These foods and their consumption have further spread from high-income to low-income countries, and with them, there has been a concomitant increase in associated diseases [2,3]. The spreading of the Western diet has also led to a rise in metabolic syndrome and obesity cases, which are now considered an epidemic [4].

Metabolic syndrome is defined as the presence of at least three of the following conditions: abdominal obesity, high blood pressure, hyperglycemia, hypertriglyceridemia, and low high-density lipoprotein (HDL) levels. Metabolic syndrome is one of the major risk factors for the development of cardiovascular disease (CVD) and type 2 diabetes (DM 2). About 25% of the U.S. adult population has metabolic syndrome, with a higher incidence among racial and ethnic minorities [5]. It is well-known in the literature that there is a close correlation between insulin resistance, metabolic syndrome, and prediabetes [6]. In fact, the incidence of metabolic syndrome is often associated with obesity and DM2. According to a survey conducted in 2015, 604,000,000 adults and 108,000,000 children globally were obese. From 1980 to the present, the prevalence of obesity has increased globally, and has doubled in 73 countries. The rate of increase is highest for childhood obesity [7]. This evidence shows that obesity is no longer a disease of affluence. The greatest increase in the prevalence of obesity in males between 25 and 29 years of age has occurred in lower-income countries. In recent years, the prevalence of obesity has increased from 1.1% (in 1980) to 3.85 (in 2015). Between 1990 and 2015, there was a 28.3% increase in the global rate of BMI-related deaths [8]. According to WHO, in 2014, 8.5% of adults had diabetes. Diabetes was the leading cause of 1.5 million deaths in 2019, and 48% of all deaths related to diabetes occurred before 70 years old. There has been an estimated 460,000 deaths from diabetic kidney disease, and hyperglycemia is responsible for 20% of deaths from CVD [9]. Between 2000 and 2019, diabetes-related mortality increased by 3%, regardless of age, with a 13% rise in low- and middle-income countries. However, the probability of dying from cardiovascular disease, cancer, chronic respiratory disease, or diabetes (the four major noncommunicable diseases) decreased by 22% globally between 2000 and 2019 [10].

### 1.1. Western Diet and Inflammation

Ultra-processed foods (UPFs) represent major cornerstones of the Western diet. The nutrition literature defines UPFs as pivotal elements of unhealthy dietary regimes. In high-income countries, UPFs already represent the most widely purchased and ingested foods, but they are rapidly gaining ground in developing countries as well. This is mainly due to their greater accessibility compared to traditional, “wholesome” foods [11,12]. UPFs have been defined as “industrial formulations composed entirely or predominantly of substances extracted from food (e.g., oils, fats, sugars, starch, and proteins), derived from food components (e.g., hydrogenated fats and modified starch), or synthesized in the laboratory from food substrates or other organic sources (e.g., flavor enhancers, colorants, and various food additives used to make the product hyper-pathetic)” [13]. Habitual ingestion of UPFs has been found to be associated with several chronic diseases. This evidence supports the role of dietary food quality, as assessed by the “extent and purpose of industrial processing” (as defined by NOVA), in the context of health care [14]. Zinöcker et al. conducted a study arguing that the Western diet is pro-inflammatory. The proinflammatory state promoted by UPFs would result from quantitative and qualitative changes in the gut microbiome. Zinöcker et al. hypothesized that UPFs create a preferential selection medium in the gut for pathogenic commensals associated with the development of inflammatory diseases [12]. Two major European cohort studies (Srour et al., Rico-Campà et al.) have reported favorable associations between UPFs, CVD, and all-cause mortality [15,16]. Srour et al. conducted a 5-year study on 105159 patients, concluding that higher consumption of UPFs was associated with an increased risk of CVD and cerebrovascular disease [8]. Rico-Campà et al. followed 19 899 participants for 15 years, concluding that high and regular consumption of UPFs (>4 servings per day) was found to be an independent risk factor (62%) for all-cause mortality. Each additional serving of UPFs resulted in an 18% increase in all-cause mortality [9]. The link between the WD and chronic diseases (diabetes, metabolic syndrome obesity, atopic dermatitis, and psoriasis) can be mainly explained by the altered diversity of the gut microbiome [17] and inducing a pro-inflammatory state [18]. Various studies have shown that diet is a major contributor to alterations in the diversity of the gut microbiome. More and more studies in the literature hypothesize an association between the microbiome, diet, and diseases related to chronic low-grade inflammation, such as atopic dermatitis [19,20,21]. A diet high in animal protein increases *Bacteroides* spp., *Alistipes* spp., and *Bilophila* spp. while decreasing *Lactobacillus* spp., *Roseburia* spp., and *Eubacterium rectale*, impairing the biodiversity of the gut microbiome [22,23,24,25]. Zhang et al. conducted a study on mice on a high-fat diet, finding a significant reduction in *Enterococcus* spp. [26]. Singh R. P. et al. conducted a similar experiment, finding comparable changes in the composition of the intestinal microbiota. This study confirmed the high presence of Proteobacteria and Firmicutes in subjects on a high-fat diet. They also detected species such as *Escherichia* spp., *Klebsiella* spp., and *Shigella* spp. in higher amounts in the group that followed a high-fat diet [27,28].

### 1.2. Dietary Trigger in Atopic Dermatitis

Atopic dermatitis (AD) is a chronic inflammatory skin disease, characterized by recurrent itchy lesions, causing a significant deterioration in patients’ quality of life. AD mostly shows an early childhood onset, typically between 3 and 6 months of age, and is often associated with other atopic disorders such as asthma, allergic rhinitis, and food allergies [29,30]. Environmental triggers, including dietary exposure, are believed to play a role in the pathogenesis of atopic dermatitis, increasing the risk of developing this disease. It is widely known in the literature that the gut microbiome plays a crucial role in the development of atopic dermatitis by regulating the maturation of the immune system through the interaction between the microbiome and the host, especially in the early years of life [31,32,33]. Short-chain fatty acids produced by the gut microbiome, including butyrate, propionate, and acetate, play an important role in inflammatory diseases, such as AD, thus explaining the association between dietary nutrition, the microbiome, and the skin immune system [34,35]. Khan et al. reviewed the current literature on the role of dietary habits, vitamin and mineral supplementation, and probiotics in the treatment and prevention of atopic dermatitis. They reported that a diet rich in fruits and vegetables may have some benefit in the treatment of AD, possibly due to flavonoids, and they indicated prebiotics as helpful in the prevention of AD in some children [36]. Schlichte et al. also wrote a review on dietary supplements for the treatment of atopic dermatitis. They focused on those containing probiotics, prebiotics, vitamin D, fish oil, Chinese herbal medicine (CHM), evening primrose oil (EPO), and borage seed oil (BO). However, they noted a considerable variability in studies; thus, due to limited evidence of efficacy in clinical trials, they do not currently recommend CHM, EPO, or BO for AD [37]. Trikamjee et al. focused on prenatal and perinatal nutritional and dietary interventions in the primary prevention of atopic dermatitis, suggesting the necessity of long-term follow-up studies to determine the true benefit of prenatal and early life dietary and nutritional interventions as a primary prevention strategy for AD [38].

## 2. Case Report

We report a case of a 14-year-old male boy who presented with a 6-year history of eczematous skin lesions in various parts of the body (face, arms, neck, and hands), suggestive of atopic dermatitis. He was also suffering from allergic asthma, disturbed sleep, and irritability. He has long been treated with both topical steroids and calcineurin inhibitors with minimal benefit. On clinical evaluation (C.E.), an itchy, eczematous eruption was seen predominantly on the mediolateral aspect of the leg shaft bilaterally, with some small lesions on both forearms and both pinnae. The face was also affected, with typical atopic facies. The affected parts excoriated and exuded a thin, sticky discharge after scratching (EASI score = 61.7; IGA score = 3; DLQI score = 21). There was aggravation at night and improvement with wet wraps. He reported that these symptoms impact his quality of life; he no longer goes out with friends, and has stopped playing sports and going to the pool. Evaluations were performed and clinical examinations revealed type I obesity (BMI = 33.2), a waist-to-hip ratio of 1, and fasting hyperglycemia (blood glucose = 112 g/dL). This placed the diagnosis as metabolic syndrome. His dietary regimen was Western-style, rich in sweet drinks, snacks, and convenience foods, and low in grains and vegetables. After enrollment, analyses were performed and Dupilumab, a monoclonal antibody directed against IL-13 and 4, was chosen. After 8 weeks of treatment (first follow-up visit), the C.E. revealed partial improvement of lesions in the lower limbs, with a reduction in erythema, lichenification, and itching (EASI score = 12.9; IGA score = 2), but the patient still deprived himself of a social life and sports activities, which had a significant impact on his quality of life (DLQI score = 19). We decided to continue dupilumab therapy, adding topical steroids as needed, and re-evaluate him at 18 weeks. After 18 weeks of treatment, the gravity scores dropped (EASI score = 0; IGA score = 0; DLQI score = 1), with complete resolution of active lesions and absence of symptoms (itching and insomnia). On C.E., the only evidence was the persistence of hyperpigmented outcomes on the upper limbs, bilaterally. Evaluations revealed weight reduction (BMI = 25) and fasting euglycemia (blood glucose = 98 g/dL). The patient reported that a week after the first follow-up visit, he consulted a nutritionist, who prescribed a regimen based on the Mediterranean diet, rich in vegetables, fruit, and wholegrains. After the nutritionist’s assessment, our patient reported that he strictly followed the prescribed diet.

## 3. Discussion

The Mediterranean diet is characterized by high intake of fruits, vegetables, and cereals, and lower intake of meats. Table 1 shows the dietary recommendations from the Mediterranean Diet Foundation. There are several systematic reviews in the literature evaluating the association between adherence to the Mediterranean diet and cardio-metabolic outcomes (weight loss and euglycemia). Esposito et al. [39] and Nordmann et al. [40] conducted a randomized trial (on 16 patients and 6 patients, respectively), evaluating the association between weight loss and diet. The results were consistent with our case, with great reductions in BMI, blood pressure (BP), and fasting glucose. The benefits of a healthy diet go far beyond weight loss and euglycemia. The Mediterranean diet has been shown to regulate inflammasome activity, with therapeutic implications in skin, systemic, and neurodegenerative inflammatory diseases. Almanza-Aguilera et al. conducted a study on 34 patients with risk factors for CVD. Adherence to a Mediterranean dietary style reduced neuroinflammation by activating TREM-1 (Triggering Receptor Expressed on Myeloid Cells 1) and the cholecystokinin/gastrin signaling pathway. This influenced the expression of pro-inflammatory cytokines and enzymes, T-cell activating receptors, nuclear factor kappa β/inflammasomes, and cell cycle regulators [41]. The Mediterranean diet (MED-Diet) has also been implicated in reducing LDL cholesterol (LDL-C) levels. The foods underlying the MED-Diet contain phytosterols, which help reduce LDL-C through the inhibition of cholesterol absorption by gut villi [42], and by blocking proprotein convertase-9 (PCSK9), which has a pivotal role in LDL-C degradation [43]. Mediterranean foods are also rich in vitamin D, whose active form (25-hydroxy-cholecalciferol) plays a role in human immunity. The relationships between vitamin D and Toll Like Receptor (TLR) have been highlighted in the literature. This suggests an action of vitamin D in the context of innate immunity and different susceptibility to infections and autoimmune diseases, such as AD [44].

## 4. Conclusions

The correlation between health and adherence to balanced diets has always been a subject of study. Our experience shows how using a therapeutic weapon such as monoclonal antibodies can benefit from the support of a euglycemic diet such as the Mediterranean diet, whose benefits go beyond simply reducing BMI and laboratory values. Our review is intended to be a prompt to highlight the need for further studies on dietary regimens and inflammatory skin diseases.

## Figures and Tables

**Table 1:** Dietary recommendations from Mediterranean Diet Foundation [45].

Foods	Servings
Olive oil	Every meal
Vegetables	≥2 servings per meal
Fruits	1–2 servings per meal
Wheat and grains	1–2 servings per meal
Dairy	2 servings/day
Nuts	1–2 servings/day
Seafood and fish	≥2 servings/week
Eggs	2–4 servings/week
Chicken	2 servings/week
Red meat	<2 servings/week
Sugary foods and drinks	<2 servings/week

## Data Availability

Data is unavailable due to privacy.
